# High sensitivity and specificity in fetal gender identification in the first trimester, using ultrasound and Noninvasive Prenatal Screening (NIPS) in twin pregnancies, a prospective study

**DOI:** 10.1186/s12884-023-06133-z

**Published:** 2023-11-22

**Authors:** Ran Svirsky, Adi Sharabi-Nov, Tal Sagi, Hamutal Meiri, Orenstein Adi, Nadav Kugler, Ron Maymon

**Affiliations:** 1 Department of Obstetrics and Gynecology, Genetic Unit, Samson Assuta Ashdod University Hospital, Ashdod, Israel; 2 Faculty of Health Sciences, Ben-Gurion University of the Negev, Be’er Sheva, Israel; 3 Department of Obstetrics and Gynecology, Shamir (Assaf Harofeh) Medical Center, Zerifin, Israel; 4 Department of Statistics, Ziv Medical Center, Safed and Tel Hai Academic College, Tel Hai, Israel; 5 PreTwin Screen Consortium and TeleMarpe Ltd, Tel Aviv, Israel; 6 Sackler Faculty of Medicine, Tel Aviv University, Tel Aviv, Israel

**Keywords:** Gender, Ultrasound, Cell-free DNA, Twin pregnancy, CRL- Crown Rump Length, Y chromosome

## Abstract

**Introduction:**

Determination of the fetal gender in the first trimester is important in twin pregnancy cases of familial X-linked genetic syndromes and helps determine chorionicity. We assessed and compared the accuracy of first-trimester ultrasound scans, and cell-free fetal DNA (CfDNA) in determining fetal gender in the first trimester of twin pregnancies.

**Methods:**

Women with twin pregnancies were recruited prospectively during the first trimester. Fetal gender was determined using both ultrasound scans and CfDNA screening. Both results were compared to the newborn gender after delivery.

**Results:**

A total of 113 women with twin pregnancies were enrolled. There was 100% sensitivity and specificity in Y chromosome detection using CfDNA.

Gender assignment using ultrasound in any first-trimester scans was 79.7%. Accuracy level increased from 54.2% in CRL 45-54 mm to 87.7% in CRL 55-67 mm and 91.5% in CRL 67-87 mm. Male fetuses had significantly higher chances of a gender assignment error compared to female fetuses, odds ratio = 23.574 (CI 7.346 - 75.656).

**Conclusions:**

CfDNA is highly sensitive and specific in determining the presence of the Y chromosome in twin pregnancies in the first trimester. Between CRL 55-87 mm, ultrasound scanning offers a highly accurate determination of fetal gender in twin pregnancies.

What’s already known about this topic?

• The accuracy of fetal gender determination in the first trimester in singleton pregnancies is well established

What does this study add?

• Above CRL of 55 mm, ultrasound is an accurate modality for gender assessment in the first trimester of twin pregnancy

• In this cohort, non-invasive prenatal screen (NIPS) had a sensitivity and specificity of 100% in the determination of Y chromosome

## Introduction

The determination of the fetal gender is often performed due to parental request and for various medical reasons such as: in families that are carriers of X-linked disorders, Duchenne muscular dystrophy, and fragile X syndrome [1], andin cases where there is a question regarding the fetal phenotype and the development of the external genitalia [2].

In cases of fetuses with congenital adrenal hyperplasia (CAH), early gender assignment and antenatal treatment with corticosteroids can prevent the masculinization of the female fetus [3].

Specifically in twin pregnancies, the determination of fetal gender can also help in the evaluation of chorionicity and labeling of the fetuses [4]. Ongoing technological progress, including high-resolution probes for abdominal and vaginal ultrasound scanning, contribute to routine first-trimester ultrasound examinations between 11 weeks + 0 days to 13 weeks + 6-day gestation and has long become an established part of antenatal care [5]. This first-trimester scan is now a common practice in fetal evaluation and gender assessment during the first trimester [6].

Sonographic determination of the fetal gender in the first trimester is performed by mid-sagittal probe plane and assessing the angle between the genital tubercle and the body axis. Fetuses with assigned male gender display a cranially directed tubercle and a higher angle; in female fetuses, the tubercle is pointed caudally with a shallow angle [7].

Another method for detecting fetal gender in the first trimester is a non-invasive prenatal screen (NIPS) using circulating free fetal DNA in the maternal blood. Gender assignment by NIPS is performed using either real-time PCR or Y chromosome read counting. The NIPS test assigns fetal gender based on whether Y chromosome material is detected or not [8]. The American College of Medical Genetics and Genomics (ACMG) recommends the use of NIPS as a first tire for screening for fetal aneuploidy, including sex chromosome aneuploidies (SCA). This approach is adopted in the Netherlands. Most genetic laboratories report fetal gender in a singleton pregnancy and the detection of the Y chromosome in twin pregnancies, upon parental request or medical needs [9, 10].

In the current study we aimed to ultrasonographical assess the accuracy of fetal gender determination during 11-14 week’s gestational age.

We further subdivided gestational age in three different cutoff of crown rump length (CRL) CRL 45-54 mm, CRL 55-67 mm and CRL 67-87 mm, and assessed the gender assignment accuracy in each group.

A secondary aim was to determine the sensitivity and specificity of Y chromosome detection using NIPS in these twin pregnancies.

## Materials and methods

This was a prospective study; we included all pregnant women aged eighteen and over, carrying two live fetuses with CRL that was measured between 45 -84 mm. All women gave their written informed consent. Excluded were women with triplet pregnancies that were reduced to twins, and cases of vanished twin. Women who subsequently lost one fetus or underwent twin reduction to singleton due to fetal defects were included. All precipitants underwent first (11-13 weeks), second (20-22 weeks), and third (28-32 weeks) trimesters scan with a complete anatomical evaluation included the determination of the fetal gender and blood drawing for other pregnancy complications. They also add additional evaluation by ultrasound in 15–16, 24-26, and 34-36 weeks. Monochorionic twins were evaluated every two weeks. Delivery and genetic results about gender outcome were retrieved from the hospital medical records of the delivery clinic and the neonatal records and, when necessary, from interviews with patients if they delivered in other hospitals.

### Gender determination during the first trimester

Sonographic assessment of fetal gender was carried out by two experienced sonographers, either transabdominal or trans vaginally by examining the genital tubercle in a midsagittal plane with the fetus horizontal (parallel) to the probe in a supine position. The angle of the genital tubercle was then assessed and compared to an imaginary horizontal line through the lumbosacral skin surface. As previously published by Efrat et al.,^7^ a male gender was assigned in cases where the sonographer estimated that the angle was higher than 30°, and female if the phallus was parallel or less than 30°.

Assessing for the presence of the Y chromosome: During the first-trimester scan, all women were offered NIPS screening for the common trisomy and the presence of the Y chromosome. Once signing on the additional annex to the informed consent requesting this test, blood samples were drawn and submitted for tests using either Invitae® or Medicover Genetics ® NIPS test for Twin Pregnancies. Since the blood draw for the NIPS test was taken on the same day as the gender assignment scan, but the NIPS results were informed weeks later. The sonographers were blinded to the results of the NIPS.

Fetal gender was subsequently ascertained in most cases by delivery records or telephone interviews. In the few cases that didn’t deliver, first-trimester gender ascertained was compared to fetal gender determined by second and third-trimester scan.

### Statistical analysis

The data were analyzed using SPSS software version 28 (IBM). P-value of 5% or less was considered statistically significant. For descriptive statistics, the categorical variables are presented as frequencies (n) and percentages, and the continuous variables are presented as medians and interquartile ranges [IQR]. For the inferential statistics, differences between groups for the continuous variables were examined using a Mann–Whitney non-parametric test. Relationships between groups and the categorical variables were calculated using Chi-square tests or the Fisher exact test (Table 1). Univariate logistic regression models, Odds Ratio (OR) and 95% confidence intervals (95% CI) were calculated to examine the association between birth gender and the ender defined by the US at the 1^st^ trimester, as stratified by group of CRL (Table 2).

**Table 1:** Maternal and pregnancy characteristics among the cohort study population

Characteristic	All (*n* = 113)	DCDA (*n* = 98)	MCBA (*n* = 15)	*p*
**Enrolment**				
Median Maternal age, years (IQR)	34.1 (30.2–37.2)	34.3 (30.2–37.3)	32.6 (29.9–35.2)	NS
Median Gestational age, wks (IQR)	12.1 (11.6–12.9)	12.1 (11.6–12.7)	12.4 (11.6–13.0)	NS
Median BMI, kg/h^2^ (IQR)	24.5 (22.0–28.6)	24.9 (22.0–29.3)	23.9 (21.1–26.1)	NS
Ethnicity n (%)				
Caucasian	108 (95.6)	94 (95.9)	14 (93.3)	NS
Arab	5 (4.4)	4 (4.1)	1 (6.7)	
CRL				
Twin 1	60.5 (53.0–69.0)	60.0 (53.0–69.0)	63.4 (51.9–69.0)	NS
Twin 2	59.9 (51.0–69.3)	59.0 (51.0–68.0)	65.8 (50.0–75.0)	NS
Conception				
Spontaneous	54 (47.8)	40 (40.8)	14 (93.3)	< 0.001
In-vitro fertilization	42 (37.2)	41 (41.8)	1 (6.7)	
Ovulation induction, AID, AIH, ICSI	17 (15.0)	17 (17.3)	0 (0)	
Nulliparous, n (%)	44 (38.9)	39 (39.8)	5 (33.3)	NS
Median fetal fraction (IQR)	14.0 (10.0–17.0)	13.5 (9.7–16.5)	16.0 (14.0–17.0)	NS
**Delivery**^**a**^				
Median Gestational age, wks (IQR)	36.5 (34.9–37.3)	36.9 (35.1–37.3)	34.6 (32.7–36.1)	0.048
Mode of delivery, n (%)				
Spontaneous	19 (23.5)	18 (24.7)	1 (12.5)	NS
Other	62 (76.5)	55 (75.3)	7 (87.5)	
The delivery outcome, n (%)				
Two fetuses born	85 (85.0)	76 (85.4)	9 (81.8)	NS
One fetus born	10 (10.0)	10 (11.2)	0 (0)	
Loss of entire pregnancy	5 (5.0)	3 (3.4)	2 (18.2)	
Birth weight				
Twin 1	2358 (2025–2645)	2401 (2075–2695)	1700 (1400–2540)	NS
Twin 2	2360 (2068–2592)	2375 (2070–2605)	2142 (1670–2545)	NS

Results are presented as n (%) or median (interquartile range- IQR). *BMI* body mass Index, *AIH* Artificial Insemination, *AID* intrauterine insemination with donor sperm, *ICSI* Intra cytoplasm sperm injection

^a^13 ongoing pregnancies were not included

**Table 2:** Accuracy of gender determination at first trimester according to birth’s gender, CRL levels and Y chromosome test

	Birth’s gender n (%)		Sensitivity of gender determination		PPV of gender determination		Accuracy of gender determination	OR (95% CI)
	Male	Female	Male	Female	Male	Female	n / N (%)	
US at 1^st^ trimester								
Male	61 (68.5)	9 (9.7)	68.5	90.3	87.1	75.0	145 / 182 (79.7)	20.3
Female	28 (31.5)	84 (90.3)						(8.0–45.2)
**CRL 45–54**								
Male	6 (25.0)	4 (16.7)	25.0	83.3	60.0	52.6	26 / 48 (54.2)	1.7
Female	18 (75.0)	20 (83.3)						(0.4–6.9)
**CRL 55–67**								
Male	30 (85.7)	3 (10.0)	85.7	90.0	90.9	84.4	57 / 65 ([1])	54.0
Female	5 (14.3)	27 (90.0)						(11.8–247.6)
**CRL 68–8**4								
Male	23 (88.5)	1 (4.8)	88.5	95.2	95.8	87.0	43 / 47 (91.5)	153.3
Female	3 (11.5)	20 (95.2)						(14.8–1593.6)
**Y chromosome**^**a**^							95 / 95 (100)	

*US* ultrasound, *CRL* crown rump length, CRL range – weeks 11, 12, 13 according to Fetal Medicine Foundation scale (https://fetalmedicine.org/research/pregnancyDating). N- Sample size of accuracy group, N – group’s total sample size, %—total accuracy of gender determination. *OR* odd’s ration, *CI* confidence interval

^a^Success in determining the gender using the Y chromosome test (Yes = accuracy was successful when the Y chromosome was found in cases of twins of M/M, M/F, F/M (M- male, female) or after reduction/one embryo loss and only one newborn – M, and when there was no Y chromosome in the cases of F/F or F. No = Y chromosome was found in cases of F/F or F

### Ethics approval

The study was approved by the Institute Review Board (IRB) with the signed Trial # 0043–20-ASF, followed by authorization by Israel Ministry of Health # 202,016,632.

This study is part of Era PerMed JCT-2019–061 – the ERAPERMED project with th title: “Develop a multi-disciplinary approach for personalized prenatal diagnostics and care for twin pregnancies” [11].

## Results

### Cohort characteristics

**Fig. 1:**
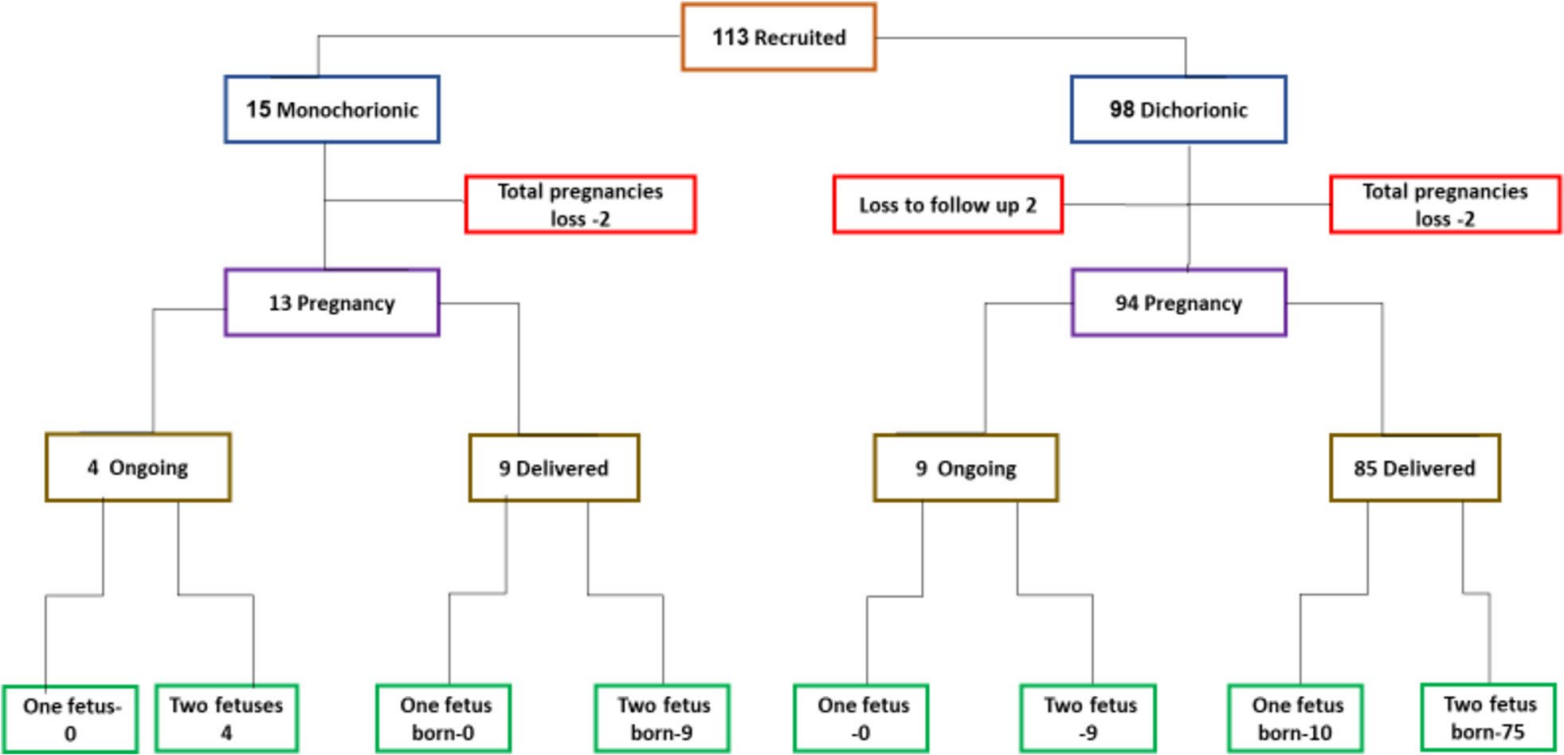
Recruitment flow chart

A total of 113 fetuses from twin pregnancies were enrolled from December 2020 to March 2023. Of these, 98 were from dichorionic diamniotic (DCDA) pregnancies and 15 from monochorionic diamniotic (MCDA) pregnancies (Fig. 1).

All these features correspond to the general characteristics of our Twin Clinic population [11, 12].

NIPS was considered accurate in cases of Y chromosome-positive when at least one of the fetuses was a male fetus and in cases of Y chromosome negative, when both of the fetuses were females. There was 100% sensitivity and specificity in Y chromosome detection using CfDNA for both monochorionic and dichorionic twin pregnancies regardless of the CRL in both Invitae’s and NIPD genetics kits.

As for the first-trimester scan: For all cases, the accuracy in gender assignment in the first trimester was 79.7%. The longer the length of the CRL, the more accurate was the geder determination. For every elevation of 1 mm in CRL length, the chances for error were reduced by a factor of 0.826. Above CRL of 55 mm, the gendr assignment was accurate in around 90% of the examined fetuses.

Male fetuses had a significantly higher chance of a gender assignment error compared to female fetuses, odds ratio = 20.3 (CI 8–45.2). Table 2 summarizes the results of the gender assignment.

Other factors such as maternal age, parity, mode of conception, and BMI had no effect on gender assignment accuracy.

## Discussion

This study is the first prospective study to compare and report the accuracy of NIPS and ultrasound scan in determining fetal gender in the first trimester. In a previous study, Efrat et.al. demonstrated an accuracy of 93% in gender assignment in the first-trimester scan in singleton pregnancy by examination of the angle of the genital tubercle [7]. Schaefer et al. reported (in a cohort that included 502 singleton pregnancies and 31 sets of twins) that when sex assignment was possible (in 22% of their cohort they were unable to assign gender) the overall accuracy of fetal gender assignment was 94.4% [13]. While Efrat et.al. found a higher accuracy for gender determination in cases of female fetuses compared to males, Schaefer et al. couldn’t demonstrate a connection between fetal gender and gender determination accuracy [7, 13]. Currently, we have demonstrated that in twin pregnancy ultrasound in the first trimester and especially CRL of 55 mm is also an accurate tool for gender determination in twin pregnancy with an accuracy rate of 87.7% in CRL 55- 67 mm and 91.5% in CRL 67–84 mm. We also demonstrated that male fetuses have a significantly higher chance of gender misassignment when compared to female fetuses. We feel that the fact that males were “misdiagnosed” more than females is probably due to the fact that when the genital tubercle angle is flat (in cases of female fetuses), there is no dilemma regarding gender assignment. The sonographer will determine correctly that the fetus is a female. Still, when there is some angle ( in cases of both female and male fetuses, especially in early gestation), the sonographer has a dilemma, and a wrong gender assignment is more prevalent.

NIPS is an established screening tool for the major trisomies in both singleton and twin pregnancies [14]. However, there are relatively few studies addressing its role in gender assignment in twin pregnancies. Villela et al. in a retrospective study developed a model that determines fetal gender with 100% sensitivity and specificity when both twins are female, and with 98% sensitivity and 95% specificity when a male is present [15]. In our prospective study there was 100% sensitivity and specificity in Y chromosome detection using CfDNA.

Our findings support the idea that both NIPS and first-trimester scan (> CRL of 55 mm) scan are accurate methods for gender assignment in the first trimester. In certain clinical scenarios such as X-linked disorders and congenital adrenal hyperplasia (CAH), NIPS can help both the parents and their physicians in their decision regarding the necessity and timing of invasive procedures and need for medical treatment.

Although our cohort consists of 113 twins; to the best of our knowledge this is the biggest study on twins addressing this issue. All were assessed in a single center by 2 experts which enchase our results. Further multi-centers studies are welcome to confirm our results.

## Data Availability

The datasets used and/or analyzed during the current study are available from the corresponding author upon reasonable request.
